# Clinicopathological and prognostic exploratory study of primary metaplastic squamous cell carcinoma of the breast

**DOI:** 10.1371/journal.pone.0333347

**Published:** 2025-09-30

**Authors:** Fang Zhang, Chengmin Zhou, Ye Yang, Junjie Li, Yue Ma, Jie Dai

**Affiliations:** 1 Department of Pathology, Sichuan Clinical Research Center for Cancer, Sichuan Cancer Hospital & Institute, Sichuan Cancer Center, University of Electronic Science and Technology of China, Chengdu, China; 2 Department of Thoracic Oncology, Sichuan Clinical Research Center for Cancer, Sichuan Cancer Hospital & Institute, Sichuan Cancer Center, University of Electronic Science and Technology of China, Chengdu, China; 3 Department of Breast Surgery, Sichuan Clinical Research Center for Cancer, Sichuan Cancer Hospital & Institute, Sichuan Cancer Center, University of Electronic Science and Technology of China, Chengdu, China; All India Institute of Medical Sciences, INDIA

## Abstract

**Background:**

To investigate the clinicopathological characteristics of primary metaplastic squamous cell carcinoma of the breast (PMSCCB), and analyze the correlation among immuno-molecular phenotype, treatment and prognosis, in order to facilitate the subsequent precise treatment.

**Methods:**

The clinicopathological data and paraffin-embedded tissue of 26 cases of PMSCCB were collected and analysed, and the relevant literatures were also studied. Given the limited sample size collected, the results are considered a preliminary exploratory analysis.

**Results:**

Twenty-six female patients, with a mean age of 53.11 ± 10.83 years, had no previous history of squamous cell carcinoma. Grossly, the tumor exhibited a solid or cystic-solid appearance, with the maximum diameter averaging 3.73 ± 2.53 cm. Microscopically, there were 18 cases of pure squamous cell carcinoma and 8 cases of mixed type. The immuno-molecular phenotype of the sample was characterised by negative rates of ER, PR, and HER-2 at 65.4% (17/26), 92.3% (24/26) and 69.2% (18/26), positive rates of HER-2 FISH, HPV RNAscope, CK5/6, p63 and EGFR at 19.2% (5/26), 4.1% (1/24), 100% (26/26), 100% (26/26) and 96.0% (24/25), respectively. Most of the 26 patients underwent surgery and chemotherapy, with some receiving additional radiotherapy or targeted therapy. After a median follow-up of months (range, 9–134 months), 17 cases had no recurrence, 8 cases had distant metastasis and 1 case was lost to follow-up.

**Conclusions:**

PMSCCB occurs exclusively in females, with a mean age of onset of 53.11 ± 10.83 years, and 46.1% of the patients were premenopausal. It is characterized by high expression of CK5/6, p63, and EGFR, which can aid in diagnosis. There is low expression of ER, PR, and HER-2, which may offer opportunities for targeted and endocrine therapies. Anthracyclines combined with cyclophosphamide or/and taxanes may prove effective in chemotherapy treatment. p16-positive expression does not necessarily indicate high-risk HPV infection, and the expression of p53, p16 and EGFR is not correlated with prognosis. However, distant metastasis and GATA3 expression are significant factors influencing the prognosis of PMSCCB.

## Introduction

Squamous cell carcinoma often occurs in the skin, oral cavity, oesophagus, cervix and other parts of the body, but primary metaplastic squamous cell carcinoma of the breast (PMSCCB) is extremely rare [1,2], with an incidence rate of 0.1% to 0.67% [3]. The clinical manifestations are qualitative hard and poor range of motion, which are inadvertently found in most patients, and the tumor is often large when found and difficult to distinguish from other breast malignancies [4]. Currently, there is no unified standard for the treatment of PMSCCB, as most studies on PMSCCB have been published in the form of case reports, resulting in limited data [5]. Therefore, it is necessary to summarize the pathological features, immunophenotype and clinical characteristics of this rare clinical case to improve the understanding and management of this disease.

Human epidermal growth factor receptor 2 (HER2), a well-acknowledged proto-oncogene involved in the genesis and progression of breast cancer, inhibits apoptosis, promotes proliferation, enhances the invasiveness of tumor cells, and facilitates angiogenesis and lymphangiogenesis of tumors. Breast cancer with overexpression of HER2 progresses rapidly, has a short chemotherapy remission period, shows poor response to endocrine therapy, and has low disease-free survival and overall survival rates. Meanwhile, HER2 amplification serves as the main reference indicator for the treatment of breast cancer with targeted drugs, for example, Herceptin [6]. The expressions of proteins such as p16 and p53 have been verified to be associated with the occurrence and development of malignant tumors. Mutant p53 can regulate the cell cycle and upregulate the expressions of oncogenes [7]. Research has confirmed that Human Papilloma Virus (HPV) infection is related to the occurrence and development of cervical cancer, ovarian cancer, and breast cancer [8]. p16 protein is an alternative detection marker for HPV infection in cervical squamous cell carcinoma. However, in breast invasive ductal carcinoma, there is a significant negative correlation between the inactivation of the tumor suppressor gene p16 and the enhanced activity of telomerase. Those with loss of p16 protein expression have enhanced telomerase activity, vigorous tumor proliferation, and a poor prognosis [8]. Nevertheless, the relationship between p16 protein expression and HPV infection in PMSCCB remains undefined, and the expression significance of HER2, p53, EGFR, etc. in PMSCCB also requires further exploration. Given the limited sample size, this study aims to provide a preliminary exploration, laying the foundation for further research.

## Materials and methods

### Materials

Twenty-six patients diagnosed with PMSCCB in Sichuan Cancer Hospital from 01/07/2013 to 01/08/2025 were collected, including 18 cases of pure squamous cell carcinoma (PSCC) and 8 cases of mixed metaplastic carcinoma with squamous cell component (MMSC) [9,10]. The proportion of the squamous cell component in MMSC ranged from 40% to 80%. The data were accessed for research purposes from August 1, 2025, to August 14, 2025. Each diagnosis was independently confirmed by two pathologists while excluding metastases from other parts of squamous cell carcinoma. Moreover, all the lesions collected in this study originated from the breast, with metastatic breast cancer in the axillary lymph nodes, chest wall, and other areas being excluded. Simultaneously, paraffin-embedded tissue specimens from the patients were systematically collected and subjected to Hematoxylin and Eosin staining, immunohistochemical analysis, and molecular detection. In addition, their clinical and pathological characteristics, as well as treatment-associated data, were comprehensively organized, and a long-term follow-up study was carried out.

### Ethics

All the retrospective analyses was approved by the Management Committee for Scient of Sichuan Cancer Hospital (SCCSMC-012025023). The study was developed in compliance with the principles of the Declaration of Helsinki and the relevant requirements of the national ethics. And the requirement of patient informed consent was waived under a protocol approved by the Management Committee for Scient of Sichuan Cancer Hospital. Members of the research team were unable to access any sensitive information that could directly or indirectly identify individual participants during or after the study. All data was anonymized before analysis.

### Methods

All specimens were fixed in 10% neutral buffered formalin, dehydrated through a graded ethanol series, cleared in xylene, embedded in paraffin, sectioned at 4 μm thickness, and stained with Hematoxylin and Eosin (H&E). Immunohistochemical staining was performed using the Roche Benchmark XT and Dako Autostainer Link 48 automated platforms. Antibodies for ER, PR, and HER2 were sourced from Ventana Medical Systems, while antibodies for p63, CK5/6, p53, GATA-3, p40, p16, EGFR, and Ki-67 were obtained from Fuzhou Maixin Company. The staining protocol followed standard procedures, including deparaffinization, antigen retrieval using a citrate buffer, and incubation with primary antibodies at appropriate dilutions. The sections were then incubated with a secondary antibody and enzyme complex, followed by color development with DAB and counterstaining with hematoxylin. HER2 gene amplification was evaluated using Fluorescence in Situ Hybridization (FISH) with a HER2/TOP2A/CSP17 multicolor probe (Guangzhou Anbiping Medical Technology Co.LTD). Tissue sections (4 μm) were deparaffinized, rehydrated, retrieved and subjected to protease digestion. The probe was hybridized overnight at 37°C, followed by post-hybridization washes. Nuclei were counterstained with DAPI. Positive ER and PR was defined according to the 2020 guidelines [11]. HER2 was assessed according to the 2023 guidelines [12].

HPV RNA was detected using the RNAscope HPV HR18 multi-subtype combined detection kit (Beijing Zhongshan Jinqiao Company), targeting 18 high-risk HPV subtypes. Tissue sections (4 μm) were deparaffinized, rehydrated, retrieved and treated with protease. The probe was hybridized according to the manufacturer’s protocol, and signals were amplified using the RNAscope 2.5 HD Detection Reagents. Nuclei were counterstained with hematoxylin. Positive HPV RNA expression was indicated by brown dots within the nuclei or cytoplasm.

### Statistical analysis

Data were analyzed using the software SPSS27.0 (IBM Corp., Armonk, NY, USA). For numeric data, results were reported as median values ± standard deviation (SD). The Fisher’s exact test was used to compare categorical values and the missing data of 1–2 cases were directly deleted. Kaplan-Meier survival curves and the Log-rank test were used to analyze survival prognosis and there were 1–3 cases of missing data, which were imputed using the mean value. P < 0.05 was considered statistically significant.

## Results

### Clinical features

All the 26 patients with PMSCCB were female, with an average age of 53.11 ± 10.83 years (range, 30–59 years) and had no skin surface ulceration and nipple depression. But, the final pathological results showed that two cases involved the nipple and dermis, and one case involved the nipple. The maximum diameter of the mass ranged from 0.8 to 9 cm (mean 3.73 ± 2.53 cm). Among most gray solid matter, 14 cases had cystic areas, and there was no history of squamous cell carcinoma in other parts. The majority of patients underwent modified radical mastectomy and were clinically staged as AJCC Stage II. However, there were no significant differences in the incidence of tumors between the left and right breasts, or in relation to menopausal status and lymph node metastasis. The specific clinicopathologic features and surgical methods are shown in Table 1.

**Table 1:** Clinicopathological features of PMSCCB.

Clinicopathological features	Unit	Values
Age, mean ± SD	Years	53.11 ± 10.83 [30 − 59]
Gender	N	
Male		0
Female		26
Menopausal status	N	
Postmenopausal		14
Premenopausal		12
Tumor site	N	
Right breast		12
Left breast		14
Surgical method	N	
Modified radical mastectomy		17
Total mastectomy		1
Total mastectomy + Lymph node exploration		8
Largest diameter of the primary tumor	CM	3.73 ± 2.53[0.8 − 9]
Presence of metastatic lymph nodes	N	
Yes		14
No		12
AJCC stage	N	
I		2
II		16
III		7
IV		1
Histological type	N	
Pure squamous cell carcinoma		18
Mixed with other types of breast cancer		8

### Microscopic observation

**Fig 1:**
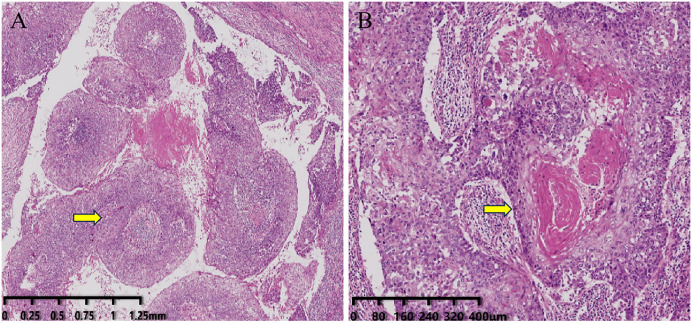
HE Staining of Tumor Cells. A: Cystic changes in squamous cell carcinoma of the breast under the microscope, with tumor cells growing in papillary form within the cyst (As shown by the arrow), visible fibrous vascular core, and the papilla covered by stratified squamous epithelia. The magnification factor showed on the lower left scale of the figure. B: The PMSCCB grows invasively within the fibrous stroma, showing the formation of keratinized beads (As shown by the arrow). The magnification factor showed on the lower left scale of the figure.

There were 18 cases of pure metaplastic squamous cell carcinoma, 7 cases with a small amount of non-specific invasive carcinoma (NST) and/or intraductal carcinoma, and 1 case with mesenchymal cartilage differentiation metaplastic carcinoma. The proportion of squamous cell carcinoma components in MMSC collected in this study ranges from 40% to 80%. The tumor cells grew in nests, sheets, papillae, expansively, or invasively, with large nuclei and prominent nucleoli, accompanied by keratinization, and visible keratin pearl formation. In some cases, the cystic structure area showed focal cystic wall tumor cell nests infiltrating growth, similar to the morphology of high-grade angiosarcoma-like or acantholytic squamous cell carcinoma. In some cases, central cystic cavity formation is observed (14/26). Among them, there were 9 cases of high-medium differentiation, 9 cases of medium differentiation, 6 cases of medium-low differentiation, and 2 cases of low differentiation. (Fig 1)

### The results of immunohistochemistry and molecular detection

**Fig 2:**
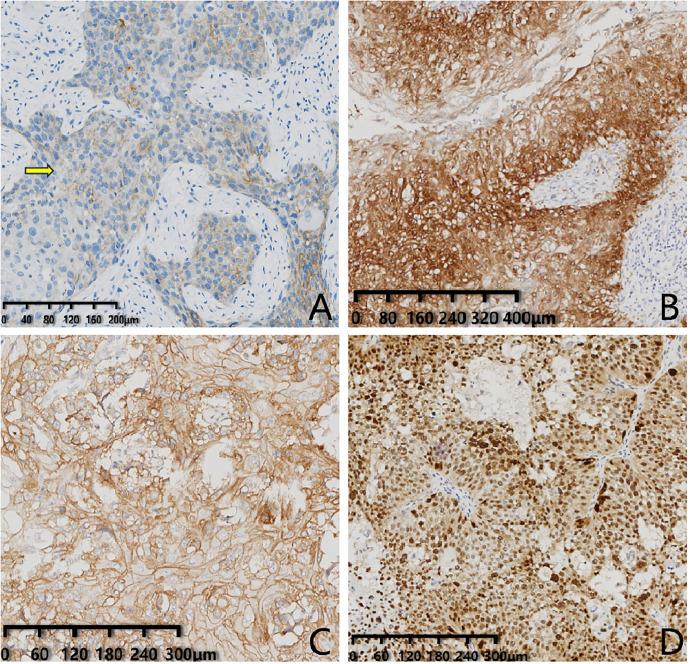
IHC Staining of Tumor Cells. A: PMSCCB shows weak-moderate intensity staining of HER-2 (En Vision) in >10% of tumor cells, with complete membrane staining. Immunohistochemistry suggests HER-2 (2+). The magnification factor showed on the lower left scale of the figure. B: Immunohistochemical staining (En Vision) of p16 in PMSCCB shows diffuse cytoplasm positive staining. The magnification factor showed on the lower left scale of the figure. C: The EGFR immunohistochemical staining (En Vision) of PMSCCB cells exhibits diffuse brown staining of the cell membrane. The magnification factor showed on the lower left scale of the figure. D: The p53 immunohistochemical staining (En Vision) of PMSCCB cells demonstrates diffuse nuclear positive staining, indicating positive expression of the p53 mutant type. The magnification factor showed on the lower left scale of the figure.

**Fig 3:**
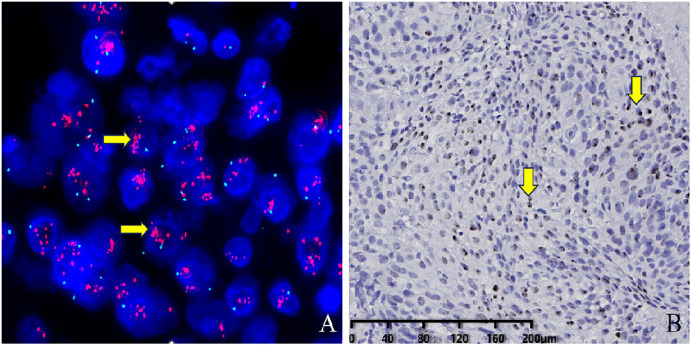
In Situ Hybridization Staining of Tumor Cells. A: Cases of HER-2 (2 + /3+) PMSCCB exhibit clustered signals on FISH, indicating HER-2 FISH amplification (+). 1000 × magnification. B: HPV RNAscope test of PMSCCB shows diffuse brownish particles in the cytoplasm, suggesting HPV RNAscope (+) and the presence of high-risk HPV virus infection. The magnification factor showed on the lower left scale of the figure.

As the subject of this study was squamous cell carcinoma, the results of the experiments involving mixed carcinoma were focused on the squamous cell carcinoma component. Molecular Immuno-phenotypes: ER (17 cases negative, 6 cases with low expression, 3 cases positive) with a negative rate of 65.4% (17/26), PR (24 cases negative, 1 case with low expression, 1 case positive) with a negative rate of 92.3% (24/26), HER2 (0, 18 cases; 1 + , 1 case; 2 + , 4 cases; 3 + , 3 cases) (Fig 2A) with IHC 2+ and above were subjected to FISH testing, with five showing amplification. The HER2 FISH amplification rate was 19.2% (5/26, Fig 3A). Among the 16 cases that were negative for both ER and PR, the distribution of HER2 status was as follows: 0 (10 cases), low expression (2 cases), and amplification (4 cases). Therefore, the proportion of triple-negative breast cancer was 38.5% (10/26). The Ki67 expression rate was 3%−95% (with an average of 44%) and the positive rates of CK5/6, p63, EGFR, p40, GATA-3, and p16 were 100% (26/26), 100% (26/26), 96.0% (24/25; Fig 2C), 80.7% (21/26), 66.7% (16/24), and 45.8% (11/24; Fig 2B), respectively. The mutation rate of p53 was 70.8% (17/24; Fig 2D). The HPV RNAscope test showed only one case with infection of high-risk HPV subtype (HPV positive infection rate was 4.1% (1/24), Fig 3B).

### Treatment and prognosis

According to the 8th edition of the American Joint Committee on Cancer (AJCC), there were two cases in stage I, sixteen cases in stage II, seven cases in stage III and one case in stage IV. All patients underwent surgical treatment: eight patients underwent total mastectomy with lymph node exploration, one patient underwent total mastectomy alone, and seventeen patients underwent modified radical mastectomy. There were fourteen cases of axillary lymph node metastasis resulting in a lymph node metastasis rate of 53.8%. Only one patient had a family history of breast cancer, and there was no history of malignancy at other sites.

**Fig 4:**
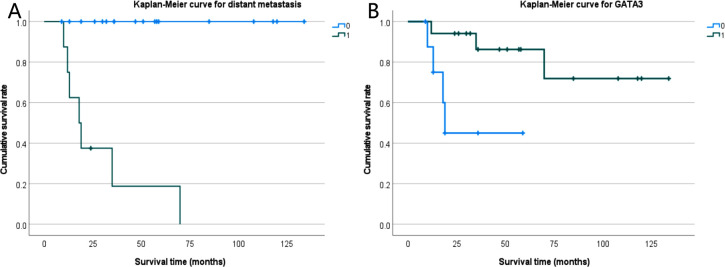
Kaplan-Meier curve for distant metastasis and GTATA3. A: Kaplan-Meier curve for distant metastasis; 0-No distant metastasis, 1-Distant metastasis, p < 0.001. B: Kaplan-Meier curve for GATA3; 0- Negative expression of GATA3, 1- Positive expression of GATA3, p = 0.017.

Approximately 38.5% of the cases in this group were triple-negative breast cancers. Preoperatively, 9 cases received neoadjuvant chemotherapy, 2 cases received neoadjuvant chemotherapy plus immunotherapy, 1 case received chemotherapy plus dual-targeted therapy, and 1 case received endocrine therapy. Postoperatively, 9 cases received chemotherapy, 5 cases received chemotherapy plus radiotherapy, 2 cases received chemotherapy plus endocrine therapy, 2 cases received chemotherapy plus targeted therapy, and 1 case received targeted therapy alone. The specific treatment regimens and drug names are detailed in Table 2. Anthracyclines were the main chemotherapy regimen, combined with cyclophosphamide or/and taxanes, and the chemotherapy cycle was determined according to the patient’s risk factors. All participants were followed up, except for one case. At the time of follow-up, 17 participants had no recurrence or metastasis, 8 had distant metastases (7 of whom died), and 1 was lost to follow-up. The mean follow-up time was 48.8 months (range, 9–134 months) for all participants and the median progression-free survival (PFS) time was 45.08 months (range, 9–134 months). (Table 2). We analyzed the correlation between p16, p53, EGFR expression levels and distant metastasis, survival status, clinical stage, and histological type of PMSCCB. However, our findings revealed no significant association between these three protein expressions and distant metastasis or prognosis in PMSCCB patients. Meanwhile, there was no significant correlation between p16 expression and HPV infection. (Table 3) Nevertheless, it is worth noting that distant metastasis emerged as a crucial factor influencing the prognosis of PMSCCB patients (P < 0.001). Similarly, GATA3 negativity also indicated poor prognosis. (P = 0.017, Fig 4).

**Table 2:** The clinical treatment and follow-up information of PMSCCB.

No.	Preoperative Treatment	Postoperative Treatment	Recurrence or Metastasis	Follow-up
1	FEC → T, 2 cycles	FEC → T 4 cycles followed by radiotherapy, GP 3 cycles	Right chest wall and bone metastasis, 13 months after diagnosis	Died 19 months after the diagnosis
2	No	ET → C 8 cycles, radiotherapy	No recurrence and metastasis	Survival of 85 months after diagnosis
3	TE 6 cycles	No	No recurrence and metastasis	Survival of 57 months after diagnosis
4	TAC 6 cycles	Trastuzumab	No recurrence and metastasis	Survival of 59 months after diagnosis
5	No	EC → T 8 cycles, radiotherapy	Right right cardiophrenic angle and sternum metastasis, 9 months after diagnosis	Died 13 months after the diagnosis
6	No	AC → T 8 cycles	No recurrence and metastasis	Survival of 51 months after diagnosis
7	No	EC → T 8 cycles, Trastuzumab	No recurrence and metastasis	Survival of 36 months after diagnosis
8	No	EC → T 8 cycles	No recurrence and metastasis	Survival of 118 months after diagnosis
9	No	EC → T 8 cycles	No recurrence and metastasis	Survival of 108 months after diagnosis
10	FEC 2 cycles, TEC 2 cycles	TEC 3 cycles, radiotherapy	Follow-up to 18 months after diagnosis, no recurrence or metastasis	Loss to follow-up
11	No	EC → T 8 cycles	Bone metastasis, 16 months after diagnosis	Died 70 months after the diagnosis
12	EC → T, 8 cycles	No	No recurrence and metastasis	Survival of 58 months after diagnosis
13	No	EC 1 cycle	Bilateral lung and bone metastases, 12 months after diagnosis	Died 12 months after the diagnosis
14	No	No	No recurrence and metastasis	Survival of 47 months after diagnosis
15	TEC 5 cycles	TEC 3 cycles, radiotherapy	Lung metastasis, 10 months after diagnosis	Died 10 months after the diagnosis
16	TXT 3 cycles	TXT 1 cycles	No recurrence and metastasis	Survival of 120 months after diagnosis
17	TAC 2 cycles, CTX+ e-ADM → LP for 4 cycles	CTX + e-ADM → LP for 2 cycles, endocrine therapy for 4 months, capecitabine + docetaxel for 4 cycles	Left chest, right axillary lymph nodes, left lung and bone metastasis, 12 months after diagnosis	Died 35 months after the diagnosis
18	No	TEC 6 cycles	No recurrence and metastasis	Survival of 134 months after diagnosis
19	No	EC → T 8 cycles	No recurrence and metastasis	Survival of 36 months after diagnosis
20	TCb 6 cycles, Pembrolizumab	No	No recurrence and metastasis	Survival of 26 months after diagnosis
21	Endocrine therapy	EC → T 8 cycles, endocrine therapy	No recurrence and metastasis	Survival of 9 months after diagnosis
22	ET → C 2 cycles, TCb+Trastuzumab +Pertuzumab 6 cycles	No	Liver metastasis, 11 months after diagnosis	Died 18 months after the diagnosis
23	No	TP 4 cycles	No recurrence and metastasis	Survival of 32 months after diagnosis
24	TCb+Pembrolizumab 6 cycles	No	No recurrence and metastasis	Survival of 13 months after diagnosis
25	No	TCb+Trastuzumab 6 cycles	No recurrence and metastasis	Survival of 30 months after diagnosis
26	PEC 6 cycles	No	Left chest wall metastasis, 20 months after diagnosis	Survival of 24 months after diagnosis

F: 5-Fluorouracil, E: Epirubicin, C: Cyclophosphamide, T: Docetaxel/Paclitaxel, G: Gemcitabine, P: Cisplatin, A: Doxorubicin, LP: Paclitaxel Liposome,Cb: Carboplatin

**Table 3:** The relationship between expression of P16, P53, and EGFR and clinicopathological features of PMSCCB.

Clinicopathological features	P16 (N)		P	P53 (N)		P	EGFR (N)		P
	Positive	Negative		Mutant type	Wild type		Positive	Negative	
**Lymph node metastasis**									
Yes	6	5	0.682	9	2	0.386	11	1	0.480
No	5	8		8	5		13	0	
**AJCC Staging**									
I-II	8	9	1.000	11	6	1.000	17	1	1.000
III-IV	3	4		6	1		7	0	
**Survival status**									
Survival	8	10	1.000	13	6	1.000	18	0	0.280
Death	3	3		4	1		6	1	
**Histological type**									
Pure	9	7	0.211	11	5	0.211	16	1	1.000
Mixed	2	6		6	2		8	0	
**HPV infection**									
Yes	1	0	0.458			**_**			**_**
No	10	13							

_: Statistics not performed;

Each group of data had 1–2 cases missing, which were directly deleted and not included in the statistics.

## Discussions

PMSCCB, as a rare type of breast cancer, is more common among middle-aged and elderly women, with an average age of onset exceeding 50 years. The average age of patients in this study is 53.11 years, which is consistent with literature descriptions. But, in this group of cases, there were 10 patients under the age of 50, accounting for 38.5%, with the youngest being 30 years old. This indicates that the risk of this disease may be increased in young and middle-aged women. PMSCCB is mainly cystic solid or solid mass, with an average diameter of 2.8–3.5 cm [13], which is slightly larger than that of NST (average diameter 1.9 cm). The average diameter of PMSCCB in our hospital is 3.73 cm, which is slightly larger than that described in literature. It also has been reported that the rate of axillary lymph node metastasis of PMSCCB is low, generally 25%−33%, but it is prone to distant metastasis [14,15]. In this study, the rate of axillary lymph node metastasis was 53.8%, which was lower than that of NST (63%), but higher than previously reported in the literature. The majority of PMSCCB is triple-negative breast cancer [16], but in our cohort, it only accounted for 38.5%. This may be related to the relatively high proportion of premenopausal women (46.2%, 12/26). In addition, there were 6 cases with low expression of ER and 3 cases with positive ER, accounting for 34.6%. Although most patients exhibited weak expression of ER, endocrine therapy could be considered in future attempts. Additionally, a few of PMSCCB HER2 positive (14.9%) were found, and some cases could achieve complete remission or progression-free survival after anti-HER2-targeted therapy [9,17]. The HER-2 amplification rate in this group was 19.2% (5/26), which was similar to that reported. Among them, 4 cases were treated with Trastuzumab or/and Pertuzumab. One patient died of liver metastasis, while the remaining 3 patients are still in PFS. This suggests that PMSCCB can also routinely undergo HER-2 testing to identify patients who may benefit from targeted therapy [17].

It has been reported that p63, CK5/6, and EGFR are crucial immune markers for the diagnosis of primary squamous cell carcinoma of the breast (PSCCB) [18,19]. The remarkably high positivity rates observed in this cohort for p63 (100%), CK5/6 (100%), and EGFR (96%), further demonstrate the importance of these three proteins in the diagnosis of PSCCB. Also, p40 is a subtype of p63 and is considered a highly specific marker for squamous cell carcinoma due to its higher specificity [20]. As a member of the HER family, EGFR is associated with unfavorable tumor prognosis; its expression is detected in approximately 40% of breast cancers and overexpression can predict a poor response to endocrine therapy among breast cancer patients [21,22]. In this study, only 2 patients with EGFR positive expression were treated with endocrine therapy, so we were unable to determine the effectiveness of the endocrine therapy. Meanwhile, we employed Fisher’s exact test, which revealed that EGFR expression had no significant association with the histological type, clinical stage, distant metastasis, or survival status of PMSCCB. This finding is inconsistent with previous reports [23]. Although EGFR is an important molecular target, the results of clinical trials of EGFR-targeted therapies in breast cancer have not been satisfactory. Further research into the mechanisms of EGFR in breast cancer is needed to better understand how to utilize EGFR–targeted therapies.

The p16 protein is a cyclin-dependent kinase inhibitor, and its overexpression is associated with poor prognosis in various cancers [24]. Given that p16 overexpression serves as a marker for cervical lesions and considering that HPV infection is a significant etiological factor for cervical cancer [21,25], we hypothesize that p16 overexpression might similarly function as a biomarker for HPV infection in patients with PMSCCB. In this study, a total of 26 patients were initially collected. However, 2 patients had undergone surgery at other hospitals and their samples were returned, leaving only 24 samples available for p16 protein staining and HPV RNA testing. Among these, the expression rate of p16 protein was 45.8% (11/24), while the positivity rate for HPV RNA was only 4.1% (1/24). These findings suggest that high-risk HPV infection may not play a significant role in the development of PMSCCB [26]. Additionally, there was no significant correlation between p16 protein expression and high-risk HPV infection in this cohort (P = 0.458), indicating that p16 cannot serve as a biomarker for high-risk HPV infection in PMSCCB. Moreover, p16 expression did not show a significant association with lymph node metastasis, clinical staging, survival status, or histological type (p > 0.05), which is different from the common histological types of breast cancer [8,24]. Chutong Ren’s research has indicated that the positivity rate of HPV infection in malignant breast tumor tissues is higher compared to benign and normal tissues, and HPV infection is associated with poor prognosis in patients [27]. However, due to the limited sample, more cases need to be collected for research and analysis.

p53 is a protein with a molecular weight of 47.3 kDa and is also a human tumor suppressor gene, which has the functions of inducing cancer cell apoptosis and repairing cellular genetic defects [28]. The p53 protein exhibits two primary expression patterns: wild-type and mutant-type. The wild-type p53 is significantly associated with cancer cell apoptosis, whereas the mutant form is implicated in the development of malignancy. Mutations in p53 are relatively common in breast cancer, with a positivity rate of approximately 30% to 40%. It is believed that p53 expression correlates with tumor aggressiveness, poor prognosis, and resistance to treatment [29]. In this group of patients, the expression of mutant-type p53 protein accounted for 70.8% (17/24), which is commonly seen in HPV-unrelated squamous cell carcinoma where p53 protein is typically expressed as a mutant [30]. However, using Fisher’s exact test and the Log-rank test, we found that p53 had no significant association with lymph node metastasis, survival status, clinical staging, or histological type. Nonetheless, recent advancements in research have introduced Rezatapopt, an investigational small molecule p53 reactivator that selectively binds to the Y220C-mutant p53 protein without interacting with wild-type or other mutant p53 proteins. In a case of triple-negative breast cancer, after 6 weeks of treatment with Rezatapopt, the tumor volume was reduced by 41% [31]. This indicates that primary metaplastic squamous cell carcinoma of the breast may potentially benefit from new targeted therapies in the future. However, further prospective studies are required to fully explore this potential.

Many studies suggest that PSCCB can be treated with 5-fluorouracil and cisplatin with or without anthracyclines, but other studies have also shown that such cancers are naturally resistant to fluorouracil, cyclophosphamide and anthracyclines, while sensitive to platinum-based chemotherapy [32]. The chemotherapy regimen of this group was mainly anthracyclines combined with cyclophosphamide or/and taxanes (76.9%, 20/26), 12 cases had no recurrence and metastasis (60%, 12/20), and the longest disease-free survival was 134 months. Therefore, anthracyclines combined with cyclophosphamide or/and taxanes may be effective in the treatment of PMSCCB patients without recurrence or distant metastasis. [19]. In addition, two patients received chemotherapy combined with the immune checkpoint inhibitor Pembrolizumab. As of the end of the observation period, these patients remained disease-free, with an overall survival (OS) of 13/26 months. Therefore, in the future, for patients with recurrent or metastatic disease, the combination of chemotherapy and immunotherapy could be considered. [33]

The mass of PSCCB is relatively large, and modified radical surgery might be more suitable for patients than breast-conserving surgery. However, some studies have pointed out that, especially after breast conserving surgery, radiotherapy can reduce the recurrence rate of breast cancer by half and the mortality rate by about one sixth [34]. The combination of radiotherapy and breast conserving surgery can achieve both mastectomy and cosmetic effects. Whereas, other literature suggests that the benefits of radiotherapy are not good, and neoadjuvant therapy might be effective [35,36]. In this study, 12 patients received preoperative neoadjuvant chemotherapy, 5 patients received postoperative radiotherapy, 7 patients died, the OS was 9–134 months, and 1 patient was lost to follow-up. Due to the limited number of patients and the retrospective analysis of this study, further studies are needed to determine whether neoadjuvant chemotherapy and radiotherapy can help reduce local recurrence of PSCCB.

The prognosis of PSCCB is considered to be worse than that of common NST-negative breast cancer, with a 5-year overall survival rate of only 34.5%−67.2% [34,37]. In our cohort, the 5-year overall survival rate was observed at 76.9%, which is higher than previously reported. Furthermore, among the 8 patients with distant metastasis, 7 succumbed to rapid disease progression. This group included 5 cases of pure breast squamous cell carcinoma and 2 cases of the mixed type. These findings underscore distant metastasis as a significant prognostic factor for patients with PMSCCB (P < 0.001). GATA3 is a transcription factor, and its prognostic significance may vary across different molecular subtypes of breast cancer. In this study, we found that high expression of GATA3 is associated with a better prognosis, similar to that observed in luminal breast cancer [38]. Moreover, the expression of GATA3 protein can be assessed in surgical or biopsy specimens, which may offer a basis for early clinical treatment decisions.

This study provides preliminary insights into the clinicopathological features and prognosis of PMSCCB through a limited sample, which restricts the statistical power and generalizability of the results. However, these findings still offer valuable information for future research and point to the direction for further investigation. Further validation is needed in a larger sample.

## Conclusions

In general, PMSCCB, as a rare form of breast cancer, predominantly affects middle-aged and elderly women, but it is showing a tendency towards younger age groups. Its immunophenotype frequently exhibits negative expressions of ER, PR, and HER2, with positive expressions of p63, CK5/6 and EGFR. These characteristics not only offer opportunities for targeted therapy and endocrine treatment but also aid in diagnosis. [39] The occurrence of PMSCCB is not associated with high-risk HPV infection; furthermore, a positive expression of p16 does not indicate the presence of HPV infection. The expressions of p16, p53, and EGFR proteins are not correlated with distant metastasis, survival status, clinical stage, and histological type in PMSCCB. However, distant metastasis and GATA3 expression are significant factors influencing the prognosis of PMSCCB. Anthracyclines combined with cyclophosphamide or/and taxanes may be effective in the treatment of PMSCCB patients without recurrence or distant metastasis.

## Supporting information

S1 File REMARK-20 checklists. (DOCX)
