# Supplementary material for: Clinicopathological and prognostic exploratory study of primary metaplastic squamous cell carcinoma of the breast
Source: PLoS One. 2025 Sep 30;20(9):e0333347. doi: 10.1371/journal.pone.0333347 (PMC12483215; doi:10.1371/journal.pone.0333347)
Supplement: S1 File — (DOCX) [file pone.0333347.s001.docx]

REMARK-20 checklists

| Introduction |  |  |
| --- | --- | --- |
|  | 1 | State the marker examined, the study objectives, and any prespecified hypotheses.  The marker examined:ER, PR, HER2,HPV, p63, CK5/6, p53, GATA-3, p40, p16, EGFR, and Ki-67  This study aims to investigate the clinicopathological characteristics of primary metaplastic squamous cell carcinoma of the breast (PMSCCB) and analyze the correlation among immunomolecular phenotype, treatment, and prognosis to facilitate subsequent precise treatment.  This study did not start with any initial hypotheses and aimed to draw conclusions based on observations and research findings. |
| Materials and methods |  |  |
| Patients | 2 | Describe the characteristics (e.g. disease stage or comorbidities) of the study patients including their source and inclusion and exclusion criteria  Twenty-six patients diagnosed with PMSCCB in Sichuan Cancer Hospital from 01/07/2013 to 01/08/2025 were collected, including 18 cases of pure squamous cell carcinoma (PSCC) and 8 cases of mixed metaplastic carcinoma with squamous cell component (MMSC).  Each diagnosis was independently confirmed by two pathologists while excluding metastases from other parts of squamous cell carcinoma. Moreover, all the lesions collected in this study originated from the breast, with metastatic breast cancer in the axillary lymph nodes, chest wall, and other areas being excluded.  All the 26 patients with PMSCCB were female, with an average age of 53.11±10.83 years (range, 30-59 years) and had no skin surface ulceration and nipple depression.According to the 8th edition of the American Joint Committee on Cancer (AJCC), there were two cases in stage I, sixteen cases in stage II, seven cases in stage III and one case in stage IV. |
|  | 3 | Describe treatments received and how chosen (e.g. randomized or rule-based)  Randomized |
| Specimen characteristics | 4 | Describe type of biological material used (including control samples) and methods of preservation and storage  The type of biological material used was formalin-fixed paraffin-embedded tissue, which was preserved and stored at room temperature. |
| Assay methods | 5 | Specify the assay method used and provide (or reference) a detailed protocol, including specific reagents or kits used, quality control procedures, reproducibility assessments, quantitation methods, and scoring and reporting protocols. Specify whether and how assays were performed blinded to the study endpoint  All specimens were fixed in 10% neutral buffered formalin, dehydrated through a graded ethanol series, cleared in xylene, embedded in paraffin, sectioned at 4 μm thickness, and stained with Hematoxylin and Eosin (H&E).  Immunohistochemical staining was performed using the Roche Benchmark XT and Dako Autostainer Link 48 automated platforms. Antibodies for ER, PR, and HER2 were sourced from Ventana Medical Systems, while antibodies for p63, CK5/6, p53, GATA-3, p40, p16, EGFR, and Ki-67 were obtained from Fuzhou Maixin Company. The staining protocol followed standard procedures, including deparaffinization, antigen retrieval using a citrate buffer, and incubation with primary antibodies at appropriate dilutions. The sections were then incubated with a secondary antibody and enzyme complex, followed by color development with DAB and counterstaining with hematoxylin.  HER2 gene amplification was evaluated using Fluorescence in Situ Hybridization(FISH) with a HER2/TOP2A/CSP17 multicolor probe (Guangzhou Anbiping Medical Technology Co.LTD). Tissue sections (4 μm) were deparaffinized, rehydrated, retrieved and subjected to protease digestion. The probe was hybridized overnight at 37°C, followed by post-hybridization washes. Nuclei were counterstained with DAPI.  Positive ER and PR was defined according to the 2020 guidelines. HER2 was assessed according to the 2023 guidelines.  HPV RNA was detected using the RNAscope HPV HR18 multi-subtype combined detection kit (Beijing Zhongshan Jinqiao Company), targeting 18 high-risk HPV subtypes. Tissue sections (4 μm) were deparaffinized, rehydrated, retrieved and treated with protease. The probe was hybridized according to the manufacturer's protocol, and signals were amplified using the RNAscope 2.5 HD Detection Reagents (Brown). Nuclei were counterstained with hematoxylin. Positive HPV RNA expression was indicated by brown dots within the nuclei or cytoplasm.  During the experimental process, only sample numbers were used, and no sensitive patient information was provided to ensure that the assays were performed blinded to the study endpoint. |
| Study design | 6 | State the method of case selection, including whether prospective or retrospective and whether stratification or matching (e.g. by stage of disease or age) was used. Specify the time period from which cases were taken, the end of the follow-up period, and the median follow-up time  This study is a retrospective study and, due to the rarity of the cases, stratification or matching (e.g., by disease stage or age) was not used.  Twenty-six patients diagnosed with PMSCCB in Sichuan Cancer Hospital from 01/07/2013 to 01/08/2025 were collected.The follow - up ended on August 25, 2025. The mean follow - up time was 48.8 months (9 - 134 months). |
|  | 7 | Precisely define all clinical endpoints examined  The clinical endpoints precisely defined included disease-free survival (DFS) and overall survival (OS). |
|  | 8 | List all candidate variables initially examined or considered for inclusion in models  The candidate variables initially examined or considered for inclusion in models included age, disease stage, perimenopause, treatment type, histological type, distant metastasis, survival status, ER, PR, HER2, HPV, p63, CK5/6, p53, GATA-3, p40, p16, EGFR, and Ki-67. |
|  | 9 | Give rationale for sample size; if the study was designed to detect a specified effect size give the target power and effect size  Due to the rarity of PMSCCB, the sample size was limited. This study aimed to provide preliminary insights rather than detect a specific effect size. |
| Statistical analysis methods | 10 | Specify all statistical methods, including details of any variable selection procedures and other model-building issues, how model assumptions were verified, and how missing datawere handled  For numeric data, results were reported as median values ± standard deviation (SD). The Fisher’s exact test was used to compare categorical values and the missing data of 1-2 cases were directly deleted. Kaplan-Meier survival curves and the Log-rank test were used to analyze survival prognosis and there were 1-3 cases of missing data, which were imputed using the mean value.  Due to the limited sample size, Cox regression analysis was not performed. |
|  | 11 | Clarify how marker values were handled in the analyses; if relevant, describe methods used for cutpoint determination  In this study, biomarker expression was dichotomized as positive or negative. Positive was coded as 1, and negative as 0. |
| Results |  |  |
| Data | 12 | Describe the flow of patients through the study, including the number of patients included in each stage of the analysis (a diagram may be helpful) and reasons for dropout. Specifically, both overall and for each subgroup extensively examined report the numbers of patients and the number of events  The final cohort consisted of 26 patients, with only one patient lost to follow-up in 2016. |
|  | 13 | Report distributions of basic demographic characteristics (at least age and sex), standard (disease specific) prognostic variables, and tumor marker, including numbers of missing values  All the 26 patients with PMSCCB were female, with an average age of 53.11±10.83 years (range, 30-59 years).  ER (17 cases negative, 6 cases with low expression, 3 cases positive) with a negative rate of 65.4% (17/26),  PR (24 cases negative, 1 case with low expression, 1 case positive) with a negative rate of 92.3% (24/26),  HER2 (0, 18 cases; 1+, 1 case; 2+, 4 cases; 3+, 3 cases)  FISH amplification rate was 19.2% (5/26)  The Ki67 expression rate was 3%-95% (with an average of 44%) and the positive rates of CK5/6, p63, EGFR, p40, GATA-3, and p16 were 100% (26/26), 100% (26/26), 96.0% (24/25), 80.7% (21/26), 66.7% (16/24), and 45.8% (11/24), respectively.  The mutation rate of p53 was 70.8% (17/24).  HPV positive infection rate was 4.1% (1/24). |

| Analysis and presentation | 14 | Show the relation of the marker to standard prognostic variables  Distant metastasis and GATA3 expression are significant factors influencing the prognosis of PMSCCB.  Both distant metastasis and negative GATA3 expression are indicative of a poor prognosis.  Other biomarkers were not associated with prognosis. |
| --- | --- | --- |
|  | 15 | Present univariate analyses showing the relation between the marker and outcome, with the estimated effect (e.g. hazard ratio and survival probability). Preferably provide similar analyses for all other variables being analyzed. For the effect of a tumor marker on a time-to-event outcome, a Kaplan-Meier plot is recommended  Kaplan-Meier analysis revealed that distant metastasis was associated with a poor prognosis, whereas positive GATA3 expression indicated a favorable prognosis. Other biomarkers were not associated with prognosis. |
|  | 16 | For key multivariable analyses, report estimated effects (e.g. hazard ratio) with confidence intervals for the marker and, at least for the final model, all other variables in the model  Due to the limited sample size, key multivariable analyses were not performed. |
|  | 17 | Among reported results, provide estimated effects with confidence intervals from analysis in which the marker and standard prognostic variables are included, regardless of their statistical significance  Kaplan-Meier survival curves and Log-Rank tests were used to separately analyze the relationship between the following factors and survival prognosis:  p16: p=0.483  p53: p=0.894  HER2 FISH: p=0.677  Perimenopause: p=0.260  Maximum tumor diameter: p=0.557  Presence of cystic structure: p=0.617  TNM stage: p=0.349  Distant metastasis: p<0.001, Distant metastasis emerged as a crucial factor influencing the prognosis of PMSCCB patients.  Mixed type: p=0.708  GATA3: p=0.017, GATA3 negativity also indicated poor prognosis.  There are still some negative results that have not been shown. |
|  | 18 | If done, report results of further investigations, such as checking assumptions, sensitivity analyses, and internal validation  No further analyses were performed, such as validation of assumptions, sensitivity analyses, or internal validation. |
| Discussion |  |  |
|  | 19 | Interpret the results in the context of the prespecified hypotheses and other relevant studies; include a discussion of limitations of the study  Given the rarity of PMSCCB, our study did not start with specific hypotheses but aimed to draw conclusions based on observations and research findings.  Limitations of the Study  Sample Size: The primary limitation of our study is the small sample size, which is a consequence of the rarity of PMSCCB. This limits the statistical power of our analyses and the generalizability of our findings.  Retrospective Design: As a retrospective study, our analysis is subject to biases inherent in this design, such as selection bias and incomplete data. The lack of randomization and potential for confounding factors further limits the strength of our conclusions.  Follow-Up Period: The relatively short follow-up period may not fully capture the long-term outcomes and survival patterns of PMSCCB patients. Longer follow-up periods are needed to provide a more comprehensive understanding of prognosis.  Lack of Multivariable Analysis: Due to the limited sample size, we were unable to perform multivariable analysis to control for confounding variables. This restricts our ability to definitively establish the independent prognostic value of the markers examined. |
|  | 20 | Discuss implications for future research and clinical value  In general, PMSCCB, as a rare form of breast cancer, predominantly affects middle-aged and elderly women, but it is showing a tendency towards younger age groups. Its immunophenotype frequently exhibits negative expressions of ER, PR, and HER2, with positive expressions of p63, CK5/6 and EGFR. These characteristics not only offer opportunities for targeted therapy and endocrine treatment but also aid in diagnosis. The occurrence of PMSCCB is not associated with high-risk HPV infection; furthermore, a positive expression of p16 does not indicate the presence of HPV infection. The expressions of p16, p53, and EGFR proteins are not correlated with distant metastasis, survival status, clinical stage, and histological type in PMSCCB. However, distant metastasis and GATA3 expression are significant factors influencing the prognosis of PMSCCB.  Our study provides preliminary insights into the prognostic significance of GATA3 and other biomarkers in PMSCCB. While our findings are promising, they need to be validated in larger, prospective studies. The clinical implications of our study suggest that GATA3 could serve as a valuable prognostic marker, potentially leading to more personalized treatment approaches and improved patient outcomes. Future research should focus on validating these findings and exploring the molecular mechanisms underlying the expression of these markers in PMSCCB.  Anthracyclines combined with cyclophosphamide or/and taxanes may be effective in the treatment of PMSCCB patients without recurrence or distant metastasis. |
